# From Consensus to Practice: Implementation and Impact of the Emergency Medicine Simulation Curriculum for Pediatrics (EM ReSCu Peds)

**DOI:** 10.7759/cureus.108727

**Published:** 2026-05-12

**Authors:** Kei U Wong, Rebekah Burns, Marc Auerbach, Samreen Vora

**Affiliations:** 1 Emergency Medicine, New Jersey Medical School, Rutgers University, Newark, USA; 2 Pediatrics, School of Medicine, University of Washington, Seattle, USA; 3 Pediatrics and Emergency Medicine, School of Medicine, Yale University, New Haven, USA; 4 Emergency Medicine, School of Medicine, Yale University, New Haven, USA

**Keywords:** curriculum implementation, emergency medicine training, emergency residency training, em rescu peds, pediatric emergency medicine, pediatrics emergency, pediatric simulation, simulation-based learning (sbl), simulation in medical education, teaching in emergency medicine

## Abstract

Background

Exposure to critically ill children during emergency medicine (EM) residency is limited and variable. General EDs care for over 85% of the 36 million annual pediatric ED visits in the U.S., highlighting a training gap. To address this, the Emergency Medicine Resident Simulation Curriculum for Pediatrics (EM ReSCu Peds) was developed by more than 50 pediatric simulation experts as a free, open-access curriculum of 16 peer‑reviewed cases. Representatives from 10 national EM, pediatric EM (PEM), and simulation organizations, along with contributors from 44 institutions, created and iteratively refined each case. Case toolkits include learning objectives, critical actions, debriefing guides, and instructor resources. Using a regional‑champion strategy to support dissemination, this hybrid implementation‑effectiveness study evaluated dissemination (web analytics), implementation, and educational effectiveness (user satisfaction and feedback).

Methodology

Dissemination used a multimodal approach with an open-access website and downloadable PDF book. Regional champions promoted local implementation and collected learner and facilitator data. From July 2021 to January 2025, dissemination was assessed via website visits, PDF downloads, and case access patterns. Implementation and effectiveness were evaluated using anonymous facilitator and learner surveys administered via QR codes after sessions. Surveys assessed demographics, satisfaction using Net Promoter Score (NPS), perceived educational effectiveness on Likert scales, and barriers through open-ended responses.

Results

The curriculum PDF was downloaded 2,278 times, and the website received 11,296 views from 594 cities across 64 countries. Most‑viewed cases included supraventricular tachycardia (1,918 views), diabetic ketoacidosis (1,895), and anaphylaxis (1,836). Least‑viewed cases included pulseless electrical activity/ventricular fibrillation (681), neonatal delivery (685), and penetrating trauma (774). Surveys included 84 facilitators and 514 learners from nine U.S. institutions across four regions. Facilitators were 51.3% faculty, 25% fellows, 17.1% residents; 59.5% had PEM fellowship training and 33.8% EM training. All sessions involved EM residents; some included pediatric residents (16.7%), nurses (15.5%), and other staff (42.9%). Facilitator NPS was 72 (81.6% promoters); learner NPS was 74 (78% promoters). Case‑specific NPS ranged from 70-90. Facilitators strongly agreed that cases effectively taught basic skills (92%), advanced skills (87%), and were realistic (76%). Learners reported similarly strong agreement: 96% for basic skills, 91% for advanced skills, 84% for realism. Qualitative themes highlighted needs for trauma references (23.2%), multimedia adjuncts (18.3%), and concise facilitator guides (26.8%). In response, two‑page facilitator flowsheets, updated trauma references, optimized imaging formats, and procedural video links were added.

Conclusions

EM ReSCu Peds showed successful global dissemination and high satisfaction, supported by a targeted regional‑champion implementation model. Both groups provided NPS scores above 70, reflecting a “world‑class” educational product. Over 90% of users endorsed effectiveness in teaching pediatric resuscitation skills. Given modest survey response relative to website traffic, generalizability may be limited. Future work will focus on strengthening data capture, expanding multimedia resources, and evaluating clinical impact.

## Introduction

Pediatric training is a critical component of emergency medicine (EM) residency, with the Accreditation Council for Graduate Medical Education (ACGME) mandating that five months or 20% of emergency department encounters involve pediatric patients and include critical care for infants and children [1]. However, opportunities to care for critically ill or injured children are often limited and highly variable across programs and influenced by seasonal fluctuations in patient volume. This variability in case mix and acuity creates the potential for significant gaps and inequities in training. EM residents and graduates frequently report low satisfaction and insufficient preparedness in pediatric care, underscoring the importance of implementing structured strategies consistent with best educational practices in pediatric EM (PEM) [2,3]. The heterogeneity of pediatric illness and injury further complicates efforts to provide consistent, high-quality training. Despite proposed revisions to EM residency program requirements in 2025, the ACGME provides minimal guidance on specific pediatric content or mandatory procedures, offering programs substantial flexibility while contributing to variation in how pediatric training is structured and delivered [1].

These gaps are further underscored by national data showing more than 85% of the 36 million children seeking emergency care annually in the United States are managed in general emergency departments by emergency clinicians without additional or specialized pediatric training [4], and by EM program directors’ reports of lower confidence in graduating EM residents’ readiness to care for pediatric patients compared to adults [5]. Collectively, these factors highlight the need for structured educational strategies to ensure competence in pediatric emergency care training.

Simulation-based education (SBE) has been established as the best practice for bridging these gaps. SBE offers structured, repeated opportunities for residents to practice high-acuity, low-frequency events in a controlled environment, improving procedural success rates, confidence, and skill retention [6,7]. SBE provides a safe environment for learners to apply Kolb’s Experiential Learning Theory by doing, reflecting, theorizing, and experimenting [8]. Studies have demonstrated that integrating SBE into EM resident curricula enhances performance and objective outcomes, making dedicated SBE sessions a cornerstone of residency training [9,10].

To address the identified gaps of pediatric experience in EM training, particularly the infrequent and insufficient opportunities to manage critically ill children, the Emergency Medicine Resident Simulation Curriculum for Pediatrics (EM ReSCu Peds) was developed [11]. This consensus-driven curriculum includes 16 peer-reviewed simulation cases developed through collaboration among more than 50 pediatric simulation experts, with implementation supported by regional champions [12-15]. Representatives from 10 national EM, PEM, and simulation organizations, including the American Academy of Emergency Medicine, American Academy of Pediatrics, American College of Emergency Physicians, Council of Emergency Medicine Residency Directors, Emergency Medicine Residents’ Association, International Network for Simulation-Based Pediatric Innovation, Research, and Education, International Pediatric Simulation Society; Pediatric Trauma Society, Society for Academic Emergency Medicine, and Society for Simulation in Healthcare, created and iteratively peer reviewed all cases, with additional contributions from EM and PEM physicians across 44 institutions to build this educational resource for preparing EM residents to care for critically ill children [12]. Building on evidence that simulation-based curricula improve pediatric resuscitation skills and confidence, this study evaluates the Emergency Medicine Resident Simulation Curriculum for Pediatrics (EM ReSCu Peds) using a hybrid implementation‑effectiveness framework. The primary objective was to assess dissemination effort, defined as broad sharing through publicly accessible channels and measured by website analytics, PDF downloads, and case access patterns. The secondary objectives were to assess its implementation and effectiveness outcomes, defined as curriculum adoption within selected residency programs supported by the regional‑champion model and assessed through facilitator and learner satisfaction and post‑session survey feedback.

## Materials and methods

Curriculum design and implementation

The curriculum comprises 16 peer-reviewed simulation cases focused on high-acuity pediatric emergencies, such as trauma, cardiac, endocrine, and neurological conditions (Table 1). Each case toolkit contains a simulation scenario along with comprehensive implementation materials, including clearly defined case-specific learning objectives, a detailed scenario script, a summary of critical actions, structured debriefing guides, and supplemental teaching resources to ensure educational standardization. Dissemination efforts began with the launch of the downloadable PDF book on January 12, 2021, followed by the release of the web‑based platform in late July 2021. Dissemination efforts aimed at promoting awareness and knowledge of the curriculum included marketing through the national EM and PEM professional societies, Academic Life in Emergency Medicine (ALiEM), the PEM listserv, and the International Network for Simulation-Based Pediatric Innovation, Research, and Education (INSPIRE).

**Table 1:** EM ReSCu Peds Simulation Cases Source: Academic Life in Emergency Medicine. EM ReSCu Peds: Emergency Medicine Resident Simulation Curriculum for Pediatrics [11].

Case #	Case Title
1	Anaphylaxis
2	Cardiac Tamponade
3	Congenital Adrenal Hyperplasia & Adrenal Insufficiency Shock
4	Congenital Heart Lesion
5	Diabetic Ketoacidosis (DKA)
6	Foreign Body Aspiration
7	Multisystem Trauma
8	Myocarditis
9	Neonatal Delivery
10	Non-Accidental Trauma
11	Pulseless Electrical Activity / Ventricular Fibrillation (PEA/Vfib)
12	Penetrating Trauma
13	Pneumonia & Septic Shock
14	Status Asthmaticus
15	Status Epilepticus
16	Supraventricular Tachycardia (SVT)

In contrast to dissemination effort, which focused on broad visibility, the implementation phase centered on action and integration of the curriculum within residency programs. The implementation of the curriculum incorporated several strategies. Following initial dissemination efforts in 2021, including the PDF launch and subsequent web‑based release, a structured regional champion model was established to promote broader adoption and support local implementation of the curriculum across emergency medicine residency programs in the United States.

The United States was divided into geographic regions (Northeast, Midwest, Southeast, Southwest, West); each was assigned a regional champion responsible for supporting implementation efforts across EM residency programs within their area. These regional champions partnered with designated site champions at individual residency programs to identify local facilitators and barriers to curriculum adoption and sustainability. Regional champions provided tailored guidance, shared best practices, and assisted in troubleshooting logistical challenges.

To ensure alignment and continuous improvement, regional champions met quarterly with the Curriculum Collaborative leadership team to review progress, exchange feedback, and refine dissemination strategies. This collaborative approach fostered accountability and created a network of advocates committed to integrating pediatric simulation into EM training. Insights from these meetings were used to refine implementation supports, such as clarifying case materials, improving resource accessibility, and streamlining facilitator tools, to enhance curriculum adoption across programs. A key implementation improvement that emerged from site feedback was the need for low-barrier access to the curriculum. Several programs reported that reliance on static PDFs limited usability during preparation and delivery. In response, the team developed a free web-based, open-access platform that provided more flexible, real-time access to materials, including downloadable PDFs. The broader dissemination efforts, through webinars and professional society newsletters, further enhanced awareness and reach.

Data collection

This study was reviewed by Yale University Institutional Review Board and deemed exempt as educational research involving minimal risk and anonymous data collection (Protocol #2000027082). Participation in surveys was voluntary, and the only identifiable information collected was the respondent’s residency program or institutional affiliation.

This work represents a hybrid implementation-effectiveness study that evaluates how the curriculum was disseminated and implemented, and its perceived effectiveness. Two primary data sources were used: 1) website analytics and 2) survey responses.

These corresponded to the two components evaluated. The primary objective was to assess dissemination efforts, defined as broad sharing through publicly accessible channels and measured by uptake metrics derived from website analytics, including PDF downloads and case access patterns. The secondary objectives were to evaluate curriculum implementation and perceived effectiveness, defined as adoption within selected residency programs supported by a regional champion model and assessed through facilitator and learner satisfaction and feedback collected from post‑session surveys to evaluate the curriculum’s perceived effectiveness in practice.

Website analytics tracked PDF downloads and page views from the time of launch (July 2021) through January 2025, providing insight into global reach and case accessed patterns. Facilitator and learner surveys were administered electronically via standardized QR codes displayed at the conclusion of each simulation session. Survey completion was voluntary and anonymous, and surveys were administered consistently across all participating sites using identical instruments. Incomplete surveys were excluded from analysis. The survey instruments were investigator‑developed and aligned with case‑specific learning objectives, rather than previously validated tools (Appendices 1, 2). Participation was coordinated by site champions in collaboration with regional champions to ensure consistent deployment procedures across institutions.

Survey instruments collected demographic information, assessed satisfaction using the Net Promoter Score (NPS), and included retrospective pre-post self-assessment questions anchored to case-specific learning objectives from learners. Respondents also provided qualitative feedback regarding barriers to implementation and suggestions for improvement.

Survey responses were collected from nine distinct medical institutions across the United States, representing all major geographic regions including the Northeast, Midwest, Southwest, and West. The participating institutions included both academic medical centers and community-based residency programs.

Data analysis

Analyses were descriptive in nature and intended to evaluate dissemination patterns, implementation characteristics, and perceived educational effectiveness rather than causal educational outcomes. Descriptive statistics were used to summarize website analytics (total PDF downloads, page views, and geographic reach), case access frequency, and survey responses from facilitators and learners. User satisfaction and perceived curriculum impact were assessed using the NPS, a standardized satisfaction metric ranging from -100 to +100. NPS was calculated according to the standard methodology as the percentage of promoters (scores 9-10) minus the percentage of detractors (scores 0-6), with passives (scores 7-8) excluded from the calculation. Scores above 70 are generally considered benchmarks for a “world-class” educational product. 

Retrospective pre-post self-assessment data were analyzed to evaluate self-reported changes in perceived competence across case-specific learning objectives. Responses were categorized into a 3-point Likert scale (i.e., strongly agree, somewhat agree, and do not agree) and compared descriptively to assess trends in perceived improvement.

Qualitative data from open-ended survey responses were analyzed using thematic review to identify recurring themes related to implementation barriers, resource needs, and suggested improvements (e.g., trauma references, digital adjuncts, and facilitator summaries).

## Results

Data were collected from July 2021 through January 2025.

Dissemination outcomes

Global Reach and Patterns

**Figure 1:**
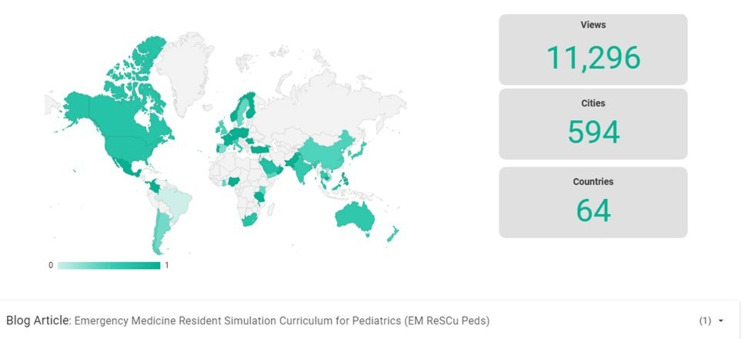
Global Reach of EM ReSCu Peds This figure illustrates the worldwide dissemination of the Emergency Medicine Resident Simulation Curriculum for Pediatrics (EM ReSCu Peds) [11]. Countries highlighted in green represent regions where the curriculum has been accessed. Key engagement metrics are displayed: 11,296 site views, 2,278 PDF downloads, and access from 64 countries. These data reflect the curriculum’s broad international adoption and sustained use since launch.* *Global Reach of EM ReSCu Peds. Data visualized using Looker Studio (Google), 2025. Image created by authors.

The primary website received 11,296 total page views from 594 distinct cities across 64 countries, reflecting broad geographic dissemination. The curriculum was downloaded as a complete PDF toolkit 2,278 times throughout the study period (Figure 1).

Case Access Patterns

Case access data revealed differential engagement across the 16 simulation scenarios (Table 2). The three most frequently downloaded cases were supraventricular tachycardia (SVT), diabetic ketoacidosis (DKA), and anaphylaxis. Conversely, the three least downloaded cases were pulseless electrical activity/ventricular fibrillation (PEA/Vfib), neonatal delivery, and penetrating trauma. Of note, the collected data represents documented downloads through the curriculum website and does not capture all implementations, particularly those conducted without formal reporting.

**Table 2:** EM ReSCu Peds Case Downloads EM ReSCu Peds: Emergency Medicine Resident Simulation Curriculum for Pediatrics

Case	Number of Uses
Supraventricular Tachycardia (SVT)	1,918
Diabetic Ketoacidosis (DKA)	1,895
Anaphylaxis	1,836
Status Epilepticus	1,473
Status Asthmaticus	1,159
Multisystem Trauma	953
Congenital Adrenal Hyperplasia & Adrenal Insufficiency Shock	952
Non-Accidental Trauma	904
Myocarditis	891
Congenital Heart Lesion	825
Pneumonia & Septic Shock	824
Foreign Body Aspiration	809
Penetrating Trauma	774
Cardiac Tamponade	755
Neonatal Delivery	685
Pulseless Electrical Activity / Ventricular Fibrillation (PEA/Vfib)	681

Effectiveness outcomes

Survey Response Characteristics

A total of 84 facilitator surveys and 514 learner surveys were analyzed from July 2021 through January 2025, for a total of 598 survey responses. Survey response patterns showed sustained engagement throughout the study period, with responses distributed across all 16 simulation cases.

Facilitator and Learner Demographics and Characteristics

Among 84 facilitator respondents, 51.3% (n=43) were faculty, 25.0% (n=21) were fellows, 17.1% (n=14) were residents, and 6.6% (n=6) were in other roles, including research associates and medical students. Regarding training background, 59.5% (n=50) completed Pediatrics-EM fellowship, 33.8% (n=28) completed emergency medicine residency, 1.4% (n=1) completed EM-Pediatrics fellowship, and 5.4% (n=5) had other training pathways. Among the 84 sessions with facilitator surveys reporting participants, 100% included EM residents, 12% (n=10) included Pediatrics residents, 11% (n=9) included nurses, and 44% (n=37) included other staff which comprised research associates and medical students. 

Simulation Delivery Modalities

Among the 84 sessions with surveys, 73 were conducted in simulation centers and 11 were conducted in situ in the clinical environment. Co-debriefers assisted in 36 of 84 sessions. Participation was required for learners in 31/84 sessions.

*User Satisfaction*: *Facilitator Net Promoter Scores*

Facilitators reported a NPS of 72 across all the cases, with 81.6% promoters (scores 9-10), 18.4% passive (scores 7-8), and 0% detractors (scores 0-6). Learner NPS ranged from 70-90 across the 16 cases. Learners reported a NPS of 74 (78% promoters-4% detractors). The NPS and the number of learners participating by case are described below in Table 3.

**Table 3:** Number of Learners and Net Promoter Scores by Case Data are presented as N (number of learners) and Net Promoter Score (NPS; range −100 to 100). All values reflect descriptive data only; no inferential statistical testing was performed for this table. Percentages are calculated using the total number of learners across all cases (N = 514).

Case	Learners, n (%)	NPS
Congenital Adrenal Hyperplasia & Adrenal Insufficiency Shock	4 (0.8%)	100
Myocarditis	1 (0.2%)	100
Diabetic Ketoacidosis	35 (6.8%)	90
Cardiac Tamponade	5 (1.0%)	90
Supraventricular Tachycardia	52 (10.1%)	86
Non‑Accidental Trauma	50 (9.7%)	86
Status Asthmaticus	21 (4.1%)	86
Foreign Body Aspiration	52 (10.1%)	85
Multisystem Trauma	34 (6.6%)	85
Neonatal Delivery	10 (1.9%)	84
Status Epilepticus	57 (11.1%)	82
Congenital Heart Lesion	47 (9.1%)	82
Penetrating Trauma	19 (3.7%)	82
Sepsis / Pneumonia (Pneumonia & Septic Shock)	58 (11.3%)	81
Pulseless Electrical Activity / Ventricular Fibrillation	25 (4.9%)	75
Anaphylaxis	44 (8.6%)	66

Perceived Educational Effectiveness

Facilitator and learner self-reported ratings of educational effectiveness are provided in Table 4 and Table 5.

**Table 4:** Facilitator Perceived Educational Effectiveness Post Survey (n=84) Data are presented as N (number of raw counts) for each response category. Descriptive statistics only; no inferential testing is performed. Percentages are calculated using the total number of facilitators across all cases (N = 84).

Statement	Strongly Agree	Somewhat Agree	Do Not Agree
Effective in teaching basic pediatric resuscitation skills	77 (92%)	6 (7%)	1 (1%)
Effective in teaching advanced pediatric resuscitation skills	73 (87%)	8 (9%)	3 (4%)
Realistic	64 (76%)	17 (20%)	3 (4%)

**Table 5:** Facilitator Perceived Educational Effectiveness Post Survey (n=514) Data are presented as N (number of raw counts) for each response category. Descriptive statistics only; no inferential testing performed. Percentages are calculated using the total number of learners across all cases (N = 514).

Statement	Strongly Agree	Somewhat Agree	Do Not Agree
Effective in teaching basic pediatric resuscitation skills	494 (96%)	15 (3%)	5 (1%)
Effective in teaching advanced pediatric resuscitation skills	468 (91%)	26 (5%)	21 (4%)
Realistic	432 (84%)	77 (15%)	5 (1%)

Qualitative Feedback and Areas for Improvement

Open-ended survey responses (n=82) identified three primary improvement themes. Evidence-based trauma management references were requested in 23.2% (n=19) of responses. Multimedia digital adjuncts, including ultrasound videos and procedural demonstrations, were suggested in 18.3% (n=15) of responses. Concise facilitator quick-reference guides were requested in 26.8% (n=22) of responses.

Specific case-related feedback included requests for: end-tidal CO2 values in vital sign displays; procedural support materials for pericardiocentesis; improved imaging file accessibility on mobile devices; pre- and post-procedure radiographs; clarification of persistent clinical findings after correct interventions; discussion of pre-intubation resuscitation in trauma; and anatomical specifications for congenital heart lesions. Positive feedback noted the value of actors in parent roles and interdisciplinary team members.

In response to feedback, two-page facilitator flowsheets were created for all 16 cases. Evidence-based trauma resuscitation references were updated. Imaging file formats were optimized for mobile device compatibility, and procedural video links were added to select cases. Table 6 presents qualitative feedback and opportunities for improvement from participants after simulation sessions.

**Table 6:** Qualitative Feedback and Improvement Opportunities From EM ReSCu Peds Users EM ReSCu Peds: Emergency Medicine Resident Simulation Curriculum for Pediatrics

Category	Theme / Area	Representative Quote or Suggested Enhancement
Feedback	Positive learner feedback	“Very useful case for emergency medicine residents in particular.”
	Interdisciplinary value	“We had nurses and a nurse facilitator participate. This interdisciplinary experience was realistic with realistic problems with dosing and IV pumps came up and were discussed.”
	Case quality	“Great cases. Might include common pitfalls for discussion points.”
	Relevance	“Cases are relevant to real-life situations.”
Improvement opportunity	Evidence-based content	Add evidence-based trauma references.
	Digital tools	Add digital tools (e.g., ultrasound videos).
	Teaching materials	Create concise facilitator summaries.

## Discussion

Pediatric resuscitation is a critical skill for EM physicians, yet clinical exposure during EM residency remains inconsistent. Beyond the EM Model of Clinical Practice, there is no current standardized PEM curriculum for EM residents, leaving many underprepared for pediatric emergency care [16]. The EM ReSCu Peds was developed through a national consensus process and disseminated as a free, open-access resource comprising 16 peer-reviewed simulation cases, designed to teach essential pediatric emergency skills. In addition to its national use, the curriculum has also been implemented internationally; a Canadian group adapted EM ReSCu Peds for delivery using the Virtual Resus Room (VRR) platform, demonstrating its feasibility for remote, team-based pediatric resuscitation training [17]. This study evaluated the dissemination effort, implementation and effectiveness outcomes of EM ReSCu Peds through a hybrid implementation-effectiveness study design.

Implementation success and global reach

Our findings demonstrate substantial international dissemination and sustained engagement, with 11,296 website views from 594 cities across 64 countries and 2,278 curriculum downloads since launch. These metrics underscore the accessibility and global relevance of EM ReSCu Peds as an educational resource for pediatric emergency care. While this study did not directly compare training modalities or methodologies across countries, the broad international uptake of EM ReSCu Peds beyond the initial target audience of North American EM residency programs suggests a shared need for accessible, standardized pediatric SBE resources across diverse training systems. The open-access format and the web-based delivery model appear to have facilitated this broad dissemination, removing traditional barriers such as cost, institutional affiliations, and geographic limitations that often restrict access to the curriculum.

The regional champion implementation strategy appears to have successfully promoted local adoption across diverse geographic regions within the United States, as evidenced by survey participation from institutions in the Northeast, Midwest, Southwest, and West. This approach, which paired regional champions with site-level facilitators, likely contributed to the curriculum’s integration into formal residency training schedules, reflected in the finding that one-third of sessions involved required learner participation. The combination of low-barrier access (free, ungated materials) and active implementation support (regional champions, quarterly collaborative meetings) represents a replicable model for disseminating educational innovations across varied training environments.

Case access patterns and implications

Case access trends revealed that higher-frequency, high-acuity conditions commonly encountered in EM practice (such as supraventricular tachycardia, diabetic ketoacidosis, and anaphylaxis) were most frequently used. This pattern suggests strong alignment between curriculum content and learner priorities, with facilitators and programs selecting cases that most directly address common gaps in resident experience. The preferential use of medical emergency cases may reflect multiple factors, potentially including clinical relevance of these scenarios to general EM practice, frequency of encounters in training, or relative ease of implementation. The specific reasons for case selection patterns were not systematically assessed in this study. 

Lower trends of PEA/ventricular fibrillation, neonatal delivery, and penetrating trauma cases may reflect several factors. These scenarios represent rare events in most general emergency department environments, potentially reducing their perceived immediacy for educators prioritizing limited SBE time. Additionally, these cases may require specialized simulation equipment or resources that create barriers to implementation. These findings indicate opportunities for targeted promotion and enhanced implementation support for these underutilized but critical scenarios.

User satisfaction and educational effectiveness

Survey data indicated high satisfaction among both facilitators and learners, with NPS meeting or exceeding the threshold of 70 generally considered indicative of a “world-class” educational product. The facilitator NPS of 72, with no detractors, reflects uniformly positive reception among simulation instructors and suggests that the curriculum successfully meets the needs of educators with varying levels of pediatric expertise. Learner NPS varied by case within a 70-90 range, with an overall NPS of 74. While all cases received strong positive endorsement (NPS ≥70), the variation suggests differential user experiences across scenarios. The factors contributing to this variation were not systematically assessed in this study. Nevertheless, even the lowest-scoring cases received strong positive endorsement, suggesting broad acceptance across the full range of scenarios.

Both facilitators and learners strongly endorsed the curriculum's educational effectiveness. Among facilitators, 92% strongly agreed that simulations effectively taught basic pediatric resuscitation skills and 87% for advanced skills. Among learners, these endorsement rates were even higher at 96% and 91%, respectively, reinforcing prior evidence supporting simulation-based education as an effective strategy for skill acquisition and confidence building in pediatric emergency care. Ratings for case realism were slightly lower, with 76% of facilitators and 84% of learners strongly agreeing that cases were realistic. This gap between perceived effectiveness and perceived realism suggests that while learners and facilitators found the simulations educationally valuable, some aspects of realism could be improved. Qualitative feedback identified specific technical enhancements that could increase perceived realism, including improved imaging accessibility, procedural support materials, and end-tidal CO2 displays in vital signs.

The findings of this study demonstrate strong global dissemination and high levels of user satisfaction and perceived educational effectiveness among facilitators and learners. While prior literature supports simulation‑based education as an effective modality for pediatric resuscitation training, the results of this study reflect user‑reported perceptions rather than objective measures of competency acquisition or clinical performance.

Facilitator and learner engagement patterns

The facilitator profile demonstrates successful engagement of both PEM specialists (59.5% PEM fellowship trained) and general EM faculty (33.8% EM residency trained), supporting the curriculum’s design goal of being accessible to instructors with varying levels of pediatric expertise. The substantial participation of fellows and residents as facilitators (42.1% combined) suggests the curriculum’s utility extends beyond faculty development to include near-peer teaching models, which may offer advantages for learner engagement and skill transfer [18]. However, variability in facilitator experience may introduce challenges, underscoring the importance of structured facilitator guides and standardized case materials; collectively, these findings suggest the curriculum supports consistent delivery by less experienced instructors.

While EM residents comprised the primary learner audience as intended (all 84 sessions included EM residents), the documented interprofessional participation (pediatric residents, nurses, respiratory therapists, pharmacists) in a substantial proportion of sessions highlights the curriculum’s flexibility for team-based training, as individual cases and learning objectives can be adapted to emphasize discipline‑specific roles, team‑based competencies, and local educational priorities while maintaining core pediatric resuscitation objectives. This interprofessional engagement aligns with principles of team-based resuscitation training, as effective pediatric resuscitation involves coordinated team performance and communication across disciplines. The curriculum’s applicability to diverse learner audiences suggests potential utility beyond EM residency training, including continuing medical education, nursing education, and interprofessional simulation programs.

User feedback and continuous quality improvement

Qualitative feedback revealed consistent themes regarding curriculum strengths and opportunities for enhancement. The most frequently cited need for evidence-based trauma management references highlights specific user concerns regarding hemorrhagic shock resuscitation and blood product administration. These requests align with documented gaps in pediatric trauma evidence, where many recommendations are extrapolated from adult data [19,20], and controversies persist regarding optimal resuscitation strategies in children [21]. The curriculum development team’s response through updated references demonstrates a commitment to evidence-based content. 

Requests for multimedia digital adjuncts (18.3% of responses) and concise facilitator guides (26.8% of responses) reflect common implementation barriers in SBE (preparation time and technical expertise). The development of two-page facilitator flowsheets directly addresses the time barrier by providing at-a-glance summaries for rapid session preparation and streamline facilitation, potentially expanding the pool of available facilitators and reducing the “activation energy” required to implement cases. The addition of procedural video links represents an initial step toward multimedia integration, though full development of interactive modules and ultrasound video libraries remains a priority for future enhancement.

The specific technical feedback regarding end-tidal CO2 displays, imaging accessibility, and scenario logic refinements demonstrates users’ deep engagement with the curriculum content and their willingness to contribute to quality improvement. This iterative refinement process, driven by end-user feedback, exemplifies principles of continuous quality improvement and participatory design in educational resource development [22,23]. The responsiveness to user needs likely contributes to sustained engagement and positive satisfaction ratings.

Future directions

Large‑scale dissemination and implementation evaluations of pediatric emergency medicine simulation curricula remain limited in the literature. Compared with prior studies that focus on single institutions or short‑term outcomes, EM ReSCu Peds represents a nationally and internationally disseminated, consensus‑driven curriculum, providing novel implementation insights within pediatric EM education.

Future work will focus on several key priorities to enhance curriculum impact and expand the evidence base. First, improving data capture mechanisms through embedded analytics, automated session logging, or integration with learning management systems would provide more comprehensive utilization of data and reduce reliance on voluntary survey completion. Second, the development and validation of objective assessment tools aligned with case-specific learning objectives would enable evaluation of competency development beyond self-reported perceptions.

Third, expanding multimedia resources, including ultrasound video libraries, procedural demonstration modules, and interactive diagnostic imaging, represents a high-priority enhancement based on user feedback. These digital adjuncts may be particularly valuable for underutilized complex cases, potentially reducing implementation barriers. Fourth, longitudinal studies examining the curriculum’s impact on actual clinical performance, patient outcomes, or practice patterns would provide valuable evidence for translational effectiveness beyond simulation performance. Exploration of the curriculum's applicability to interprofessional training, continuing medical education, and international contexts may also identify opportunities for broader impact. As the curriculum evolves, maintaining the commitment to open access, evidence-based content, and user-driven continuous improvement will be essential to sustaining relevance and effectiveness.

Limitations

This study has several limitations. The modest survey response rate relative to overall website usage (598 surveys vs. 11,296 views) raises the possibility of response bias, with more engaged or satisfied users more likely to participate and complete surveys. The true effectiveness and satisfaction across all implementations may differ from our survey-based findings. Additionally, the reliance on self-reported survey data and investigator‑developed survey instruments, which were not formally validated, limits inferences regarding objective learning or skill acquisition. 

The geographic concentration of survey responses from nine institutions, while representing diverse US regions, may not fully capture the experience of international users or community-based programs with different resource levels. Case utilization data represents documented sessions reported through voluntary survey completion and does not capture all implementations of the curriculum. The variability in curriculum implementation across sites, differences in facilitator experience, and reliance on voluntary reporting limit standardization and reproducibility. Actual utilization is likely higher than reported, but we cannot determine the magnitude of underreporting or whether underreporting varies systematically by case type, institution, or implementation quality. Additionally, the study did not assess objective educational outcomes, long‑term skill retention, or downstream clinical impact, reflecting limitations inherent to large‑scale educational implementation research. Finally, while our data spans a 3.5-year period demonstrating sustained engagement, longer-term follow-up will be important to assess curriculum durability and impact on actual clinical practice patterns.

## Conclusions

EM ReSCu Peds achieved widespread dissemination and high facilitator and learner satisfaction as a free, open-access SBE curriculum that addresses critical gaps in PEM training for EM residents. The curriculum demonstrated global reach spanning 64 countries, sustained utilization across diverse cases and institutions, and strong satisfaction ratings from both facilitators and learners. Effectiveness outcomes showed that over 90% of respondents endorsed the curriculum’s effectiveness in teaching basic pediatric resuscitation skills, with slightly lower but still strong endorsement for advanced skills. Collectively, these findings support successful dissemination and positive perceived educational effectiveness; however, objective measures of learner competency and downstream clinical impact were not assessed. Future work will focus on validating assessment tools, strengthening data capture mechanisms, and evaluating clinical outcomes.

## Appendices

Appendix 1 

**Table 7:** EM ReSCu Peds – Facilitator Survey Instrument This survey evaluates facilitator perceptions of each EM ReSCu Peds simulation session. EM ReSCu Peds: Emergency Medicine Resident Simulation Curriculum for Pediatrics, APP: advanced practice provider, NP: nurse practitioner, PA: physician assistant

Survey Item	Response Format / Options
Simulation Case Conducted	Select the simulation case facilitated today (one survey per case)
Likelihood to Recommend	0–10 scale (“How likely are you to recommend this simulation to a friend or colleague?”)
Agreement Statements	Rate agreement for each statement: • Strongly Disagree • Disagree • Neutral • Agree • Strongly Agree
Number of Participants/Learners	Numeric entry
Role / Self‑Description	• Faculty • Fellow • Resident • Nurse • APP (NP/PA) • Other (specify)
Training Path / Planned Path	• Emergency Medicine • Pediatrics • Pediatric Emergency Medicine • Combined EM‑Peds • Other (specify)
Session Components Completed	Check all that apply: • Pre‑brief • Case facilitation • Debriefing • Technical troubleshooting • Assessment of learners • Other (specify)
Institution / Residency Program	Text entry
Open‑Ended Feedback	Free‑text response: “Please provide specific feedback to help us improve this case.”

Appendix 2 

**Table 8:** EM ReSCu Peds – Participant (Learner) Survey Instrument: Anaphylaxis Case Sample This survey assesses learner perceptions and changes in confidence following the EM ReSCu Peds Anaphylaxis simulation. EM ReSCu Peds: Emergency Medicine Resident Simulation Curriculum for Pediatrics, MS: medical school year, PYG: post-graduate year

Survey Item	Response Format / Options
Likelihood to Recommend	0–10 scale (“How likely are you to recommend this simulation to a friend or colleague?”)
Pre‑Session Agreement Statements	Rate agreement BEFORE the session: • Strongly Disagree • Disagree • Neutral • Agree • Strongly Agree
Post‑Session Agreement Statements	Rate agreement AFTER the session: • Strongly Disagree • Disagree • Neutral • Agree • Strongly Agree
Perception of the Simulation Case	“This simulation case was _______.” (Likert scale or free text, depending on item)
Key Learning Point	Free‑text: “List one key learning point from this case that you hope to apply in the future.”
Residency/Specialty Path	Select one: • Emergency Medicine • Pediatrics • Pediatric Emergency Medicine • Combined EM‑Peds • Other (specify)
Postgraduate Year / Role	• MS3/MS4 • PGY‑1 • PGY‑2 • PGY‑3 • PGY‑4+ • Fellow • Other (specify)
Institution / Residency Program	Text entry
Additional Feedback	Free‑text: “Please provide additional feedback on this case to help us improve.”
